# Lactobacillus-Dominated Cervical Microbiota Revealed by Long-Read 16S rRNA Sequencing: A Greek Pilot Study

**DOI:** 10.3390/genes17010018

**Published:** 2025-12-26

**Authors:** Despina Vougiouklaki, Sophia Letsiou, Konstantinos Ladias, Aliki Tsakni, Iliana Mavrokefalidou, Zoe Siateli, Panagiotis Halvatsiotis, Dimitra Houhoula

**Affiliations:** 1 Department of Food Science and Technology, Faculty of Food Sciences, University of West Attica, 12243 Athens, Greece; dvougiouklaki@uniwa.gr (D.V.); sletsiou@uniwa.gr (S.L.); aliki_tsak@yahoo.gr (A.T.); hlianamavrokefalidou@gmail.com (I.M.); 2 Microbiological Center Life Check, 11526 Athens, Greece; kladias@yahoo.com; 3 Medical School Sapienza, University of Rome, 00185 Rome, Italy; zoesiateli27@gmail.com; 4 2nd Propaedeutic Department of Internal Medicine, Medical School, National and Kapodistrian University of Athens, “ATTIKON” University Hospital, 12461 Athens, Greece; phal@gmail.com

**Keywords:** cervical microbiota, full-length 16S rRNA sequencing, Oxford Nanopore MinION, *Lactobacillus crispatus*, *Lactobacillus iners*

## Abstract

**Background/Objectives:** The vaginal microbiota constitutes a highly dynamic microbial ecosystem shaped by the distinct mucosal, hormonal, and immunological environment of the female genital tract. Accumulating evidence suggests that shifts in cervical microbial composition and function may influence host–microbe interactions and contribute to gynecological disease risk. Within this framework, the present study aimed to perform an in-depth genomic characterization of the cervical microbiota in a well-defined cohort of Greek women. The primary objective was to explore the functional microbial landscape by identifying dominant bacterial taxa, taxon-specific signatures, and potential microbial pathways implicated in cervical epithelial homeostasis, immune modulation, and disease susceptibility. **Methods:** Microbial genomic DNA was isolated from 60 cervical samples using the Magcore Bacterial Automated Kit and analyzed through full-length 16S rRNA gene sequencing using the Nanopore MinION™ platform, allowing high-resolution taxonomic assignment and enhanced functional inference. In parallel, cervical samples were screened for 14 HPV genotypes using a real-time PCR-based assay. **Results:** The cervical microbial communities were dominated by *Lactobacillus iners*, *Lactobacillus crispatus*, and *Aerococcus christensenii*, collectively representing over 75% of total microbial abundance and suggesting a functionally protective microbiota profile. A diverse set of low-abundance taxa—including *Stenotrophomonas maltophilia*, *Stenotrophomonas pavanii*, *Acinetobacter septicus*, *Rhizobium* spp. *(Rhizobium rhizogenes*, *Rhizobium tropici*, *Rhizobium jaguaris*), *Prevotella amnii*, *Prevotella disiens*, *Brevibacterium casei*, *Fannyhessea vaginae*, and *Gemelliphila asaccharolytica*—was also detected, potentially reflecting niche-specific metabolic functions or environmental microbial inputs. No HPV genotypes were detected in any of the cervical samples. **Conclusions:** This genomic profiling study underscores the functional dominance of *Lactobacillus* spp. within the cervical microbiota and highlights the contribution of low-abundance taxa that may participate in metabolic cross-feeding, immune signaling, or epithelial barrier modulation. Future large-scale, multi-omics studies integrating metagenomics and host transcriptomic data are warranted to validate microbial functional signatures as biomarkers or therapeutic targets for cervical health optimization.

## 1. Introduction

The microbial communities that inhabit the human body play a crucial role in determining health and disease. Specifically, the microbiota residing along the female reproductive tract have received increasing scientific attention, with the cervical microbiome emerging as a distinct and biologically important niche. The vaginal microbiota (VM) is characterized by a heterogeneous variety of microorganisms that are commonly found in cervicovaginal samples of female patients in both pathological and non-pathological conditions. Although contiguous with the vaginal environment, the cervix microbial communities reflect its unique mucosal physiology and immunological landscape. Emerging evidence suggests that shifts in cervical microbial composition may influence the persistence of high-risk human papillomavirus (HPV) infection and the progression to cervical intraepithelial neoplasia (CIN) or cancer [1,2,3,4].

Cervical microbial communities in reproductive-age women are often described through community state profiles analogous to those in the vagina, with a dominance of *Lactobacillus* species in healthy states (e.g., *L. crispatus*, *L. gasseri*, *L. iners*, *L. jensenii*) and more diverse anaerobe-rich communities in dysbiotic states [4,5].

*Lactobacillus*-rich cervical microbiota are generally associated with a lower pH, improved mucosal barrier function, and reduced risk of HPV persistence and lesion development, while depletion of *Lactobacilli* and overrepresentation of anaerobic bacteria—such as *Gardnerella*, *Prevotella*, *Sneathia*, and *Streptococcus*—have been linked to HPV infection and cervical disease severity [4,6]. Among these bacteria, *Sneathia* has emerged as one of the most prominent non-*Lactobacillus* genera linked to pathogenic outcomes; epidemiological reviews and meta-analyses frequently report its enrichment in high-grade cervical lesions and its strong association with HPV persistence, suggesting a potential co-carcinogenic or disease-modifying role within the dysbiotic cervical microenvironment [7]. Lactic acid production, bacteriocin secretion, and modulation of local immune signaling are among the proposed mechanisms through which *Lactobacillus* species may exert protective effects, although the precise molecular and host–microbe interactions remain under investigation [2,4].

Importantly, the cervical microbiome appears dynamic. Longitudinal and cross-sectional studies show that community composition may shift in response to hormonal fluctuations, sexual behavior, physiological changes, or infection. Such transitions between *Lactobacillus*-dominated and more diverse states may influence HPV acquisition, persistence, and disease progression [3,8]. Yet, the ecological and host factors that drive these shifts—whether through immune modulation, metabolic changes, or other pathways—are not fully understood.

Much of our current insight into cervical microbial communities comes from 16S rRNA gene amplicon sequencing, allowing taxonomic characterization across large cohorts [1]. More recently, multi-omics approaches (including shotgun metagenomics and metabolomics) have provided deeper insight into microbial function, diversity, and host–microbe interactions. For example, multi-omics work has linked specific bacteria (e.g., *L. iners*, *Prevotella bivia*) to metabolic pathways associated with CIN and HPV status [1]. Metabolomic profiling has also revealed key metabolite changes in cervicovaginal fluid (e.g., succinic acid) that correlate with microbial shifts and disease severity in HPV-positive women [9]. The cellular biology of HPV supports a primary role in the development of neoplasia. The viral types most closely associated with CC, particularly HPV-16 and 18, can transform cells in the culture to lose their normal growth control mechanisms. The DNA from these types integrates into host DNA.

Despite these advances, metatranscriptomic data on the cervical microbiome remain limited. Functional profiling of microbial gene expression could clarify how microbial communities modulate their activity in response to infection or host signals, and how these changes contribute to disease progression. Integrative, high-resolution studies combining community composition, gene expression, and host response may provide mechanistic understanding of how cervical microbes influence HPV persistence, cervical inflammation, and neoplastic transformation. Such work has the potential to uncover microbiome-based biomarkers for early detection of cervical disease and therapeutic strategies (e.g., probiotics or microbiome modulation) aimed at maintaining or restoring protective microbial states.

The purpose of this pilot study is primarily exploratory, aiming to assess the cervical microbiota profiles of homogenous non-pregnant reproductive-age Greek women. In addition, we investigated potential association between human papillomavirus (HPV) infection and cervical microbiota composition by determining 14 HPV genotypes in cervical samples. Through comprehensive integrative analyses, we aim to characterize the functional landscape of the cervical microbiota and uncover key microbial activities that may contribute to cervical health and disease.

## 2. Materials and Methods

### 2.1. Study Design and Cervical Samples Collection

This study was designed as a pilot exploratory analysis. A sample size of 60 cervical samples was selected to provide sufficient microbial diversity coverage for high-resolution 16S rRNA sequencing, while remaining feasible within logistical and resource constraints. Previous microbiome pilot studies have demonstrated that sample sizes of 30–100 are adequate for identifying dominant taxa and community structure patterns, particularly in low-diversity microbial ecosystems such as the cervicovaginal niche. Sixty independent cervical samples were collected from 60 women with written consent. Additionally, women who had taken antibiotics within the last three months were excluded.

#### Sample Collection

Cervical samples were collected using BD BBL Culture Swab (Becton, Dickinson and Company, Franklin Lakes, NJ, USA) for microbial analysis in a single visit. In addition, the cervical brush was inserted into the cervical canal to collect ectocervical and endocervical cells for HPV genotypes and stored at  −80 °C. Two different samples were collected for each volunteer.

### 2.2. Genomic DNA Extraction

DNA was extracted using the Magcore Bacterial automated Kit following the protocol recommended by the supplier. The concentration of each extracted DNA was measured with a Qubit 4 fluorometer (Thermo Fisher Scientific, Waltham, MA, USA).

### 2.3. DNA Amplification, Barcoding and Library Preparation and 16S rRNA Sequencing

The extracted gDNA was then prepared for prokaryotic metagenome sequencing using the 16S Barcoding Kit 0–24 (SQK-RAB204 and SQK-16S024, Oxford Nanopore Technologies, Oxford, UK), according to the manufacturer’s protocol, using 35–350 ng of the extracted gDNA per sample. The PCR reaction was performed on the full 16S hypervariable region (V1–V9) by injecting each multiplexing barcode included in the 16S Barcoding Kit 0–24 into each extracted DNA under the following conditions: initial 30 s denaturation at 98 °C (Stage 1), 25 cycles of 10 s denaturation at 98 °C, 30 s annealing at 55 °C, 90 s extension at 65 °C (Stage 2), and 5 min final extension at 65 °C (Stage 3), with NEBNext^®^ Ultra™ II Q5^®^ Master Mix (New England Biolabs, Ipswich, MA, USA) as the PCR polymerase reagent mixture. The 16S V1–V9 amplicons were subsequently purified using AMPure XP magnetic beads. The final elution of purified DNA was performed by adding 15 μL Elution Buffer. The concentration of each purified amplicon was measured with a Qubit 4 fluorometer (Thermo Fisher Scientific, Waltham, MA, USA), and the samples were pooled with a total DNA of 80 ng.

Basecalling was carried out using the Guppy agent (version 6.3.7) embedded within the EPI2ME platform (version 5.2.13, ONT), converting FAST5 files into FASTQ format. Barcode sequences were removed, and only reads with a q-score of at least 9 were retained. The resulting FASTQ files were further analyzed with Minimap2.

### 2.4. HPV Genotypes 14 Real-TM Quant

PCR was performed using Sacace Biotechnologies HPV Genotypes 14 Real-TM Quant following the protocol recommended by the supplier. Kit from Sacace Biotechnologies HPV Genotypes 14 Real-TM Quant is an in vitro Real Time amplification test for quantitative or qualitative detection and genotyping of Human Papillomavirus (16, 18, 31, 33, 35, 39, 45, 51, 52, 56, 58, 59, 66, 68).

### 2.5. Community State Type (CST) Classification

Community State Types (CSTs) were assigned based on the relative abundance profiles of dominant Lactobacillus species and anaerobic taxa, following established cer-vicovaginal microbiome classification frameworks. Samples dominated by *L. crispatus*, *Lactobacillus gasseri*, *L. iners*, *or Lactobacillus jensenii*/*Lactobacillus mulieris* were classified as CST I, CST II, CST III, and CST V, respectively. Samples characterized by reduced Lactobacillus abundance and increased representation of anaerobic or non-Lactobacillus taxa were classified as CST IV. CST assignment was performed using relative abundance thresholds and dominant taxon criteria to enable descriptive community-level comparison across samples.

### 2.6. Bioinformatics and Statistical Analysis

The reliability of the subsampled read numbers was verified. Prior to subjecting the full dataset to taxonomic analysis, three random samples from the ONT sequencing were subsampled to 30,000 reads and 100,000 reads per sample to ensure adequate read depth was achieved. The results showed negligible differences of less than 0.1% in all taxonomic levels, showing that the read depth of 30,000 reads per sample was sufficient for detecting minor constituents of the 16S. For the main taxonomic analysis, 50,000 reads per sample were used to further ensure adequate read depth. For diversity analysis, the alpha rarefaction curves of various alpha diversity parameters were checked to verify the plateau of the curves. A heatmap was generated following hierarchical clustering of samples based on Bray–Curtis dissimilarity using Ward’s linkage, after filtering low-abundance taxa (≥0.1% relative abundance in ≥10% of samples). Principal component analysis (PCA) was conducted on the same filtered dataset following data scaling to explore overall variance patterns among samples. For compositional visualization, mean relative abundances of bacterial taxa were calculated across all samples and displayed using a doughnut chart. Taxa were categorized into abundance ranges: high abundance (>80%), moderate abundance (5–20%), low abundance (1–5%), and rare taxa (<1%). The statistical analysis was performed in GraphPad Prism 10 for mac, while data visualization and graphing were performed in SRplot [10].

## 3. Results

### 3.1. Clinical and Demographic Characteristic of Samples

All analyzed specimens consisted of cervical samples collected from women of reproductive age (18–39 years). The study population displayed homogeneous demographic characteristics, with no reported gynecological disorders or significant clinical comorbidities.

### 3.2. Evaluation of Microbial Diversity

**Figure 1:**
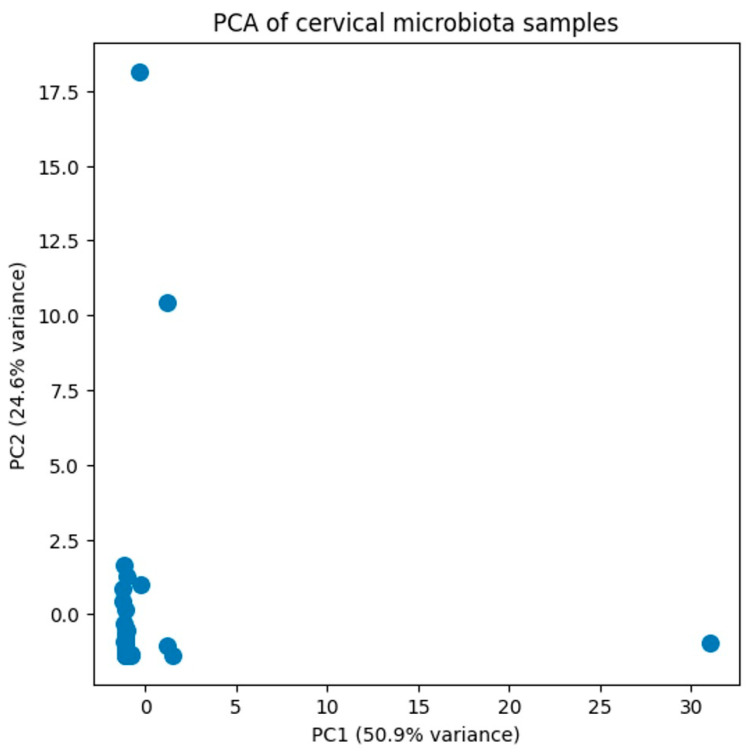
Principal component analysis (PCA) was performed on the filtered relative abundance dataset of cervical microbiota. The first two principal components (PC1 and PC2) explain 50.9% and 24.6% of the total variance, respectively. Samples are widely distributed in ordination space, indicating high inter-individual variability and the absence of distinct clustering patterns based on overall community composition.

To assess the microbial diversity within our study population, a principal component analysis (PCA) was conducted. The first two principal components (PC1 and PC2) explain 50.9% and 24.6% of the total variance (Figure 1). PCA did not reveal clear segregation of samples, indicating a high degree of inter-individual variability in cervical microbiota composition. Variance along PC1 and PC2 was largely driven by differences in dominant taxa abundance rather than discrete community groupings.

**Figure 2:**
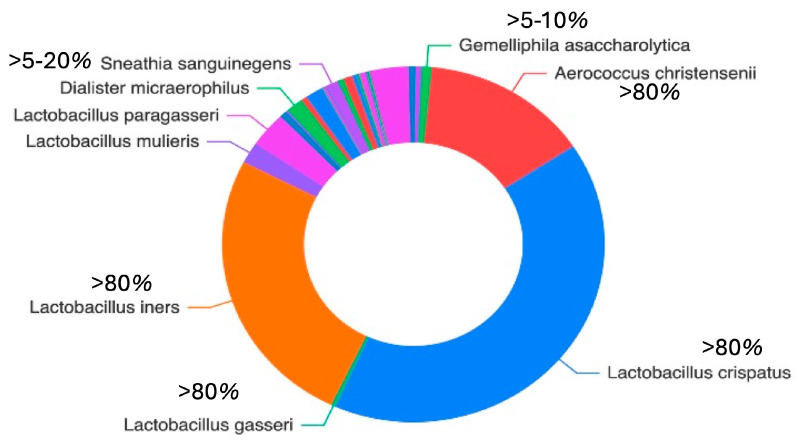
The doughnut chart illustrates the mean relative abundance of the most prevalent bacterial taxa across all samples. Taxa are grouped according to abundance ranges to improve interpretability: high abundance (>80%), moderate abundance (5–20%), low abundance (15%), and rare taxa (<1%).

Figure 2 illustrates the distribution of the detected species across all cervical samples. *Lactobacillus* spp. (notably *L. iners* and *L. crispatus*) and *Aerococcus* spp. (particularly/*A. christensenii)* were the most abundant species, together collectively accounting for more than 75% of the total microbial community. Additional species relative frequently identified included *S. maltophilia*, *S. pavanii*, *A. septicus*, *R. rhizogenes*, *R. tropici*, *R. jaguaris*, *P. amnii*, *P. disiens*, *B. casei*, *F. vaginae*, *G. asaccharolytica*, *flexneri* (Figure 2).

**Figure 3:**
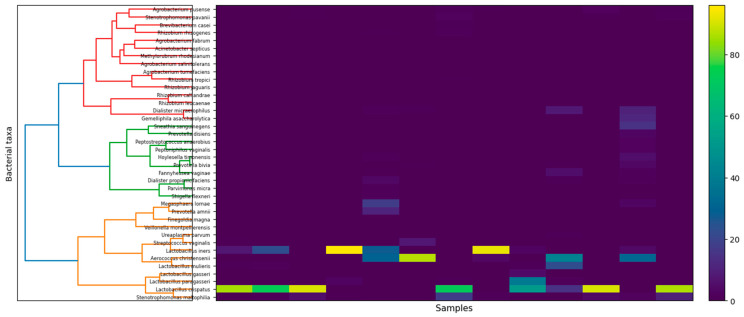
Heatmap of relative abundances of prevalent cervical bacterial taxa across samples. Samples were hierarchically clustered using Bray–Curtis dissimilarity and Ward’s linkage following filtering of low-abundance taxa (≥0.1% relative abundance in ≥10% of samples). Color intensity indicates relative abundance. Individual sample identifiers are omitted for clarity.

In addition, the hierarchical clustering heatmap (Figure 3) shows the relative abundance of dominant cervical bacterial taxa across samples. Low-abundance taxa were filtered prior to analysis (≥0.1% relative abundance in at least 10% of samples). The analysis reveals distinct community profiles driven by dominant taxa, including *Lactobacillus*-dominated and non-*Lactobacillus*-dominated microbiota structures.

## 4. Discussion

The composition of the cervical microbiota is not static but varies over time in response to hormonal status, age, sexual behavior, and environmental factors [11,12]. In our study, which included women of reproductive age, several *Lactobacillus* species—particularly *L. crispatus*, *L. iners*, *L. paragasseri* (formerly *L. gasseri*), and *L. mulieris* (formerly *L. jensenii*)—were reported to be recurrently associated with healthy microbial states and protection against infection and inflammation [13]. In addition, our sample was free of HPV according to our results on HPV genotype detection.

When interpreted within the Community State Type (CST) framework, the observed microbial profiles gain additional biological context. The predominance of CST I (*L. crispatus*-dominant) and CST III (*L. iners*-dominant) profiles in this cohort is consistent with cervicovaginal microbiome patterns reported in healthy Euro-pean populations and supports the biological plausibility of the absence of HPV-positive samples in the present study. However, given the exploratory nature of this pilot study, these observations should be interpreted with caution. Within this framework, the Lactobacillus-dominated profiles observed in this cohort primarily correspond to CST I and CST III community structures.

Among these species detected in NGS analysis, *L. crispatus* is considered the hallmark of a stable vaginal microbiome. It exerts its protective role primarily through the abundant production of lactic acid (both D- and L-isomers) and hydrogen peroxide, maintaining a low pH and directly inhibiting the proliferation of opportunistic microorganisms [14]. In the context of in vitro fertilization (IVF), a moderate and balanced abundance of *L. crispatus* appears to be favorable for pregnancy success, supporting a healthier reproductive environment [15]. In our study, *L. crispatus* was consistently identified across all cervical samples analyzed, confirming its dominant and stabilizing role within the cervical microbiota, with relative abundance of up to 99.62%. *L. iners* was also detected in every sample, exhibiting highly variable abundance levels (up to 91.07%.), reflecting its metabolic adaptability and potential involvement in transitional microbial states. At the same time, *L. iners* displays a metabolically flexible phenotype that enables persistence under fluctuating environmental conditions, yet its dominance has been associated with transitional or less stable vaginal microbiota configurations and an increased risk of bacterial vaginosis and sexually transmitted infections, like *Chlamydia trachomatis*, human immunodeficiency virus (HIV), *Neisseria gonorrhoeae* and HSV-2 [16]. In addition, *L. iners* tends to inhibit unpredictable behavior during pregnancy. Several studies have suggested that its dominance, as opposed to the more protective *L. crispatus*, may raise the risk of preterm birth, although the findings are inconsistent [16]. Overall, *L. iners* is classified as an ‘intermediate’ bacterium that does not provide consistent protection throughout pregnancy. Compared to other *Lactobacillus* species, *L. iners* possesses more complex nutritional requirements and a Gram-variable morphology. Moreover, the genome of *L. iners* encodes inerolysin, a pore-forming toxin homologous to vaginolysin from *Gardnerella vaginalis*. This feature implies that *L. iners* may encompass distinct clonal variants—some contributing to vaginal homeostasis, while others are linked to dysbiosis and disease [13]. Beyond classical Lactobacillus-dominated CSTs, transitional or mixed microbial profiles were also observed in this cohort. In this context, *A. christensenii* was detected in several cervical samples, with relative abundances up to 87.32%, indicating substantial inter-individual variability.

Previous studies have suggested that *A. christensenii* is a commensal species of the vaginal and cervical microbiota, often coexisting with *Lactobacillus* spp. in eubiotic states [13]. However, its presence at higher proportions has also been associated with transitional microbial profiles and, in some cases, with mild inflammatory responses or subclinical dysbiosis [17,18]. Recent findings reinforce its potential clinical relevance, as *A. christensenii* was found to increase in abundance among patients experiencing recurrent bacterial vaginosis following metronidazole therapy, particularly in those who relapsed [19]. Moreover, Norenhag (2024) reported higher levels of *A. christensenii* in women with cervical dysplasia compared to healthy controls, alongside increased microbial diversity and reduced *Lactobacillus* dominance, suggesting a potential link between *A. christensenii* and early cervical epithelial alterations. Furthermore, *A. christensenii* has genes related to pathogenicity, bloodstream invasion, and antibiotic resistance, which can lead to several complications, like chorioamnionitis and bacteremia. Recent findings demonstrate that *A. christensenii* can survive in the blood and cause infection [20]. Lin et al. also highlighted the need of recognizing this microorganism as a possible pathogen in pregnancy and including it into clinical evaluation of reproductive tract infections [20]. In our study, the coexistence of *A. christensenii* with *L. crispatus* and *L. iners* in most of the cervical samples may therefore represent a balanced microbial state, where *Aerococcus* species possibly contribute to mucosal defense under eubiotic conditions, but could also participate in transitional or dysbiotic shifts under altered host or environmental contexts [17,18,19].

In our analysis, *L. gasseri* was detected in low relative abundances (up to 4.77%), indicating its limited yet consistent presence within the cervical microbiota. The recently reclassified *L. paragasseri*, previously grouped within *L. gasseri*, has been identified in both vaginal and cervical samples and may contribute to epithelial protection via lactic acid production and cell adhesion, although its precise physiological function remains to be elucidated [21,22,23]. Similarly, *L. mulieris* was identified in our samples with relative abundances ranging up to 22.94%. This species, a close phylogenetic relative of *L. jensenii*, has been increasingly recognized as a commensal member of the vaginal niche, potentially supporting microbial stability and mucosal defense through the production of lactic acid and competitive exclusion of opportunistic pathogens [24,25]. One such species, *L. jensenii*, releases biosurfactants that disrupt biofilms of pathogens such as *Enterobacter aerogenes* and *Escherichia coli* [26]. In addition, a number of unexpected or low-abundance bacterial species such as: *S. maltophilia*, *S. pavanii*, *Dialister micraerophilus*, *Dialister propionicifaciens*, *A. septicus*, *R. rhizogenes*, *R. tropici*, *R. jaguaris*, *P. amnii*, *P. disiens*, *B. casei*, *F. vaginae*, *G. asaccharolytica*, *Agrobacterium pusense*, *Agrobacterium salinitolerans*, *Agrobacterium tumefaciens* have been reported in cervical microbiome studies, though the functional significance of many remains uncertain. For example, *S. maltophilia* (a member of Proteobacteria) has been detected in higher abundance in cervical intraepithelial neoplasia (CIN) samples compared with healthy controls [27]. In addition, the presence of *S. maltophilia* may be associated with persistent or recurrent vaginal discharge. This implies that even if the more prevalent causes (like candidiasis) have been treated, the presence of this pathogen may still induce or prolong symptoms [28]. Multivariate analyses in such studies also identified Rhizobium genera (family Rhizobiaceae) as independently associated with CIN [27]. Environmental or plant-related genera such as Agrobacterium and Rhizobium may reflect transient colonization, possible contamination, or low-biomass bacterial populations rather than stable, functionally active members of the cervical niche. Given the low-biomass nature of cervical samples, the detection of such taxa should be interpreted with caution, as they may reflect background environmental signals or methodological sensitivity rather than true biological colonization.

In contrast, several anaerobic taxa typically associated with dysbiotic CST IV profiles were also detected, although generally at lower relative abundances. *D. micraerophilus* (or closely related *Dialister* spp.) and *P. amnii/P. disiens* are often enriched in states of vaginal or cervical dysbiosis [29]. Additionally, recent findings highlight *D. micraerophilus* as an important contributor to the metabolic and ecological landscape of BV-associated cervical dysbiosis, complementing the established pathogenic roles of *F. vaginae* and other anaerobic taxa [30]. *F. vaginae* (formerly *Atopobium vaginae*) is well recognized bacterium as a hallmark taxon of dysbiotic vaginal communities and has been consistently associated with adverse cervicovaginal conditions [31].

As a well-established BV-associated bacterium, *F. vaginae* plays a critical role in polymicrobial biofilm formation on the vaginal epithelium, where it engages in reciprocal transcriptomic interactions with *Gardnerella* spp. and *P. bivia* [32]. Beyond its established involvement in BV, increasing evidence indicates that *Fannyhessea* contributes to broader cervicovaginal pathophysiology. In patients with persistent high-risk HPV infection and high-grade cervical intraepithelial neoplasia (CIN), members of the *Fannyhessea* genus mediate distinct metabolomic shifts associated with *Lactobacillus* depletion, epithelial barrier disruption, and mucosal immune dysregulation [33]. Furthermore, systematic reviews of cervical carcinogenesis describe non-*Lactobacillus*-dominant states enriched in *F. vaginae* as potential cofactors that may sustain chronic inflammation, modulate local immune responses, and promote viral persistence, thereby contributing to an environment permissive to neoplastic progression [34]. Other less common species such as *B. casei*, *G. asaccharolytica*, *S. pavanii*, *A. septicus*, *R. tropici/jaguaris*, and *Agrobacterium tumefaciens*/*pusense/salinitolerans*—are rarely reported in cervical microbiome cohorts. Their detection may represent very low abundance, transient exposure rather than established colonization. The biological roles of these species in the cervix are thus currently unknown. Indeed, environmental genera such as *Stenotrophomonas* and *Rhizobium* have been observed in some CIN-associated cervical microbiome studies; however, most of the well-characterized dysbiotic bacteria in this niche are anaerobes such as *Dialister*, *Prevotella*, and *Fannyhessea*. Although usually at low relative abundances, *A. septicus* (*A. septicus*) was found in a subset of cervical and endometrial samples, indicating its status as a transitory or low-biomass component of the female reproductive tract microbiota. According to previous reports, *A. septicus* is an opportunistic environmental microbe that is sometimes found in uterine or vaginal samples, especially in research using high-resolution metagenomic techniques [35,36]. Higher *A. septicus* signal intensity has been seen in pregnancies complicated by inflammation-associated preterm birth, where it appeared as part of a wider shift toward diverse, low-Lactobacillus communities, even though it is typically interpreted as a commensal or incidental taxon. Moreover, sporadic clinical observations—most notably bacteremia cases in obstetric wards—suggest that *A. septicus* may gain transient pathogenic potential under disrupted mucosal or iatrogenic conditions [35].

In a small percentage of vaginal samples, *R. rhizogenes* was occasionally found, usually at very low relative abundances, which is consistent with its classification as an environmental or low-biomass taxon within the reproductive tract. Recent high-resolution metagenomic studies have revealed that *R. rhizogenes* is present in the vaginal microbiota of pregnant people, especially in cohorts at risk for inflammation-driven preterm birth, even though it is mainly recognized as a plant-associated organism [37]. In these investigations, increased *R. rhizogenes* signal emerged in transitional community states marked by decreased *Lactobacillus* dominance and increased ecological diversity and was interpreted as a sign of wider microbial instability rather than direct pathogenicity.

Additional analyses of host–microbiome interactions likewise place *R. rhizogenes* among low-abundance taxa associated with heightened immune activation in pregnancy-related dysbiosis [18].

*R. tropici* is classified as a primarily environmental and plant-associated taxon rather than a stable component of the human reproductive tract microbiome because it was only occasionally and at very low abundances found in all the cervical samples that were examined. Despite being a well-characterized symbiotic nitrogen-fixing species in legumes, *R. tropici*’s appearance in human metagenomic datasets has typically been interpreted as incidental, reflecting fluctuations in the low-biomass community or temporary environmental contamination rather than actual colonization [38].

As a recently identified environmental species with no known function in the human reproductive system, *R. jaguaris* only occasionally and consistently appeared at trace-level abundances in the examined samples. *R. jaguaris* was first identified from legume-associated root nodules, but it has not been linked to human colonization or pathogenicity. Its infrequent discovery in cervical metagenomic datasets is typically interpreted as a low-biomass signal or temporary environmental carryover rather than a significant microbial presence [39].

*P. amnii* is a known member of the larger anaerobic community linked to both stable and transitional states of the female reproductive tract microbiome. Since its initial isolation from amniotic fluid, *P. amnii* has been associated with a variety of cervicovaginal profiles and is more common in communities with subtle inflammatory signatures and increased diversity [18]. Previous research shows that increased *P. amnii* levels may be involved in low-grade mucosal inflammation or early dysbiotic microbiome, especially in ecosystems linked to bacterial vaginosis [40,41].

According to our results, the predominance of *Lactobacillus* species in cervical samples is indicative of a balanced and eubiotic microbial environment. Their synergistic activities—acidification of the vaginal milieu, inhibition of pathogenic colonization, modulation of host immunity, and reinforcement of epithelial barrier function—highlight their crucial contribution to maintaining cervical–vaginal homeostasis and protecting against dysbiosis and infection. In addition, more targeted and high-resolution studies (e.g., using metagenomics and metatranscriptomics) throughout different populations are needed to clarify whether environmental or other species play any functional role in cervical health or disease.

The main strengths of this study include the use of long-read 16S rRNA sequencing for high-resolution taxonomic assignment and the analysis of a well-characterized cohort. Limitations include the pilot sample size, cross-sectional design, and limited statistical power, which preclude causal inference and extensive subgroup analyses. Future studies should focus on larger and more diverse cohorts and longitudinal study designs to validate these findings and to investigate temporal microbiota dynamics in relation to hormonal changes and HPV acquisition or clearance. In addition, integrative high-resolution approaches, such as shotgun metagenomics and metatranscriptomics, will be essential to elucidate the functional roles of both dominant and low-abundance taxa and to assess their potential relevance to cervical health and disease.

## 5. Conclusions

This study provides a comprehensive characterization of the cervical microbiota in non-pregnant, reproductive-age women, revealing a microbial landscape dominated by Lactobacillus species—particularly *L. crispatus* and *L. iners*—and supported by variable contributions from *A. christensenii* and several low-abundance taxa. The predominance of *Lactobacilli* reflects a generally eubiotic cervical environment, while the presence of transitional or dysbiosis-associated anaerobic genera (*Dialister*, *Prevotella*, and *Fannyhessea*) highlights the inherent ecological variability of the cervical niche. These findings reinforce the central role of Lactobacillus-driven mucosal protection and underline the complexity introduced by low-biomass or environmentally derived species whose functional relevance remains uncertain. Future research across diverse population and clinical contexts will be essential to determine whether specific microbial community patterns can serve as biomarkers or therapeutics targets for improving cervical health.

## Data Availability

The original contributions presented in this study are included in the article. The data of the current study are available from the corresponding author upon reasonable request.
